# Neostigmine to Relieve a Suspected Colonic Pseudo-Obstruction in a Burn Patient: A Case-Based Review of the Literature

**Published:** 2013-01-07

**Authors:** Abel A. Gebre-Giorgis, Ensign Joseph D. Roderique, Dane Stewart, Michael J. Feldman, Andrea L. Pozez

**Affiliations:** The Evans-Haynes Burn Center, Virginia Commonwealth University Health Systems, Richmond

## Abstract

**Objective:** Neostigmine is one of the treatment options for colonic pseudo-obstruction in the medical patient. However, experience in using neostigmine for this indication in burn patients has not been reported in the literature. We will present a case of a woman who developed colonic pseudo-obstruction during her hospital stay. When conservative management failed, neostigmine was administered with no adverse effects and resolution of the pseudo-obstruction. We will review the literature regarding the pathophysiology and treatment options for acute colonic pseudo-obstruction in burn patients. **Methods:** A 27-year-old woman with 35% total body surface area deep-partial and full-thickness flame burns. On hospital day 17, she developed a nonobstructive ileus. She failed conservative medical therapy. After consultation with colleagues in trauma surgery and a review of the literature (MeSH/PubMed/NLM), the decision was made to try neostigmine therapy rather than a surgical/procedural option such as colonoscopy. **Results:** The patient was moved to the intensive care unit and 2 mg of neostigmine was administered intravenously over 4 minutes. After 30 minutes, all abdominal examination findings had returned to baseline. No significant adverse effects were noted, and she did not redevelop abdominal distension afterward. **Conclusion:** This case report provides an alternative treatment modality in which neostigmine was used successfully in a burn patient after conservative medical treatment had failed. The authors believe that neostigmine may be a viable alternative to decompressive colonoscopy in burn patients for whom mechanical obstruction is properly excluded.

Acute colonic pseudo-obstruction (ACPO) consists of massive dilatation of the colon in the absence of mechanical obstruction. This severe form of ileus, also known as “Ogilvie syndrome,” may develop in hospitalized patients and is associated with a wide variety of medical and surgical conditions. Concomitant to colonic dilation, most patients will present with one or more of the following: abdominal pain, nausea, vomiting, and absence of flatus and stools.^1^

The differential diagnosis of colonic dilation includes the following: mechanical blockage (the most common cause), *clostridium difficile* infection, toxic megacolon, and ACPO. Before a patient can be definitively diagnosed with ACPO, the first 2 must be ruled out. Mechanical obstruction is ruled out via radiological evidence, and *C. difficile* infection must be ruled out via a stool sample, or in high-risk situations, via limited sigmoidoscopic assessment of the pseudomembranes.^2^

The most life-threatening complications in patients with ACPO are colonic perforation and ischemia. Perforation can spontaneously arise and will greatly increase morbidity and mortality. If colonic perforation occurs, patients will present with fever, abdominal tenderness, and elevated leukocytes. The risk of perforation is estimated at anywhere from 3% to 15%, with concomitant mortality as high as 50% or higher.^3^ The risk of perforation and ischemia increase substantially if cecal diameter increases more than 12 cm, or if symptoms fail to resolve within 6 days.^2^

## PREDISPOSING FACTORS IN A BURN PATIENT

In the vast majority of cases, ACPO is associated with some underlying medical condition. The most common predisposing diseases are trauma, infections (particularly those induced by gram-negative bacteria), and cardiac and neurological diseases.^1^^,^^4^

A wide variety of other predisposing factors have been implicated in the pathogenesis of ACPO. These include prolonged bed rest, high doses of narcotic medications, hypokalemia and other electrolyte and metabolic imbalances, sepsis, and surgery. As each of these factors are commonly associated with burn patients, the tendency of burn patients to develop ACPO is greater than that of the average population; a fact of which burn doctors must remain cognizant.^1^^,^^5^ In a study of more than 2703 burn patients, 0.29% went on to develop ACPO.^6^ In another study performed in the 70s, 5 patients out of 529 burn patients examined, or 1%, went on to develop ACPO.^7^

## CASE REPORT

**Figure 1:**
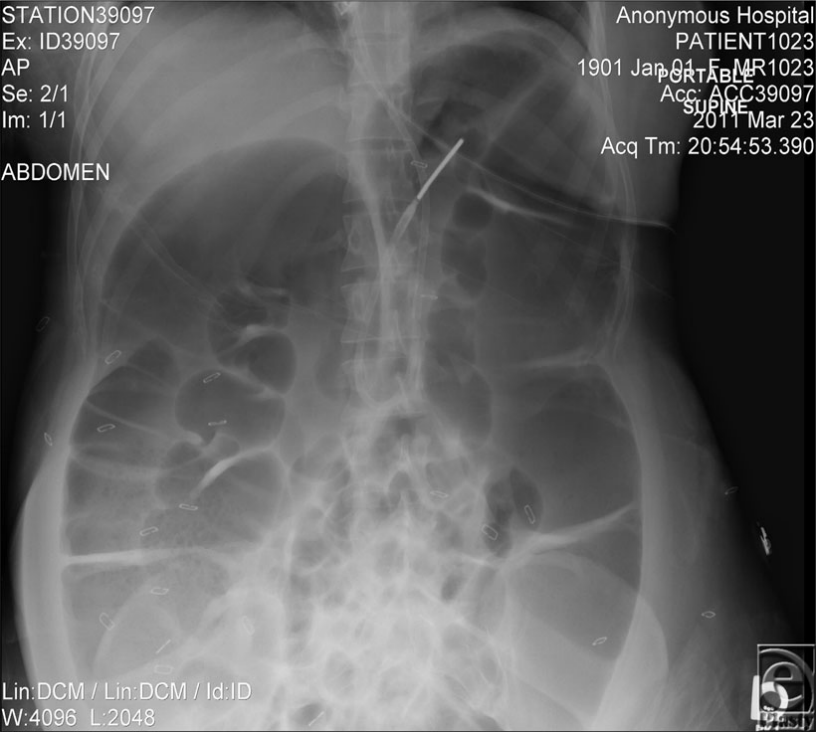
Note the marked dilatation of both the cecum and the transverse colon. Also note the presence of air down to the rectosigmoid junction, with no evidence of obstruction. Also visible in this image are the staples from the multiple skin grafts and the Dobbhoff NG tube feed catheter. All patient identifiers have been scrubbed from this image and replaced with random information.

A 27-year-old woman who is a recent immigrant of East Indian descent was admitted to our burn center with a 35% total body surface area deep-partial and full-thickness flame burn to her torso, buttocks, and circumferential bilateral upper and lower extremities. The patient underwent staged excisions, integra placement, autografting, and VAC (vacuum-assisted wound closure device) therapy. The patient began experiencing abdominal discomfort and distension on hospital day 17. She failed conservative therapy which included *nil per os*, intravenous (IV) fluid administration, nasogastric decompression, laxatives, promotility agents, stool softeners, and correction of electrolytes. On hospital day 21, despite having regular bowel movement and flatus, we noted a drastic increase in abdominal girth with associated discomfort and nausea. A plain abdominal radiograph indicated significant colonic distension, with a cecal diameter of 12 cm with associated air extending to the rectosigmoid junction (Fig 1).

After consultation with colleagues in trauma surgery and a review of the literature (MeSH/PubMed/NLM), the decision was made to try neostigmine therapy rather than a surgical/procedural option such as colonoscopy.

**Figure 2:**
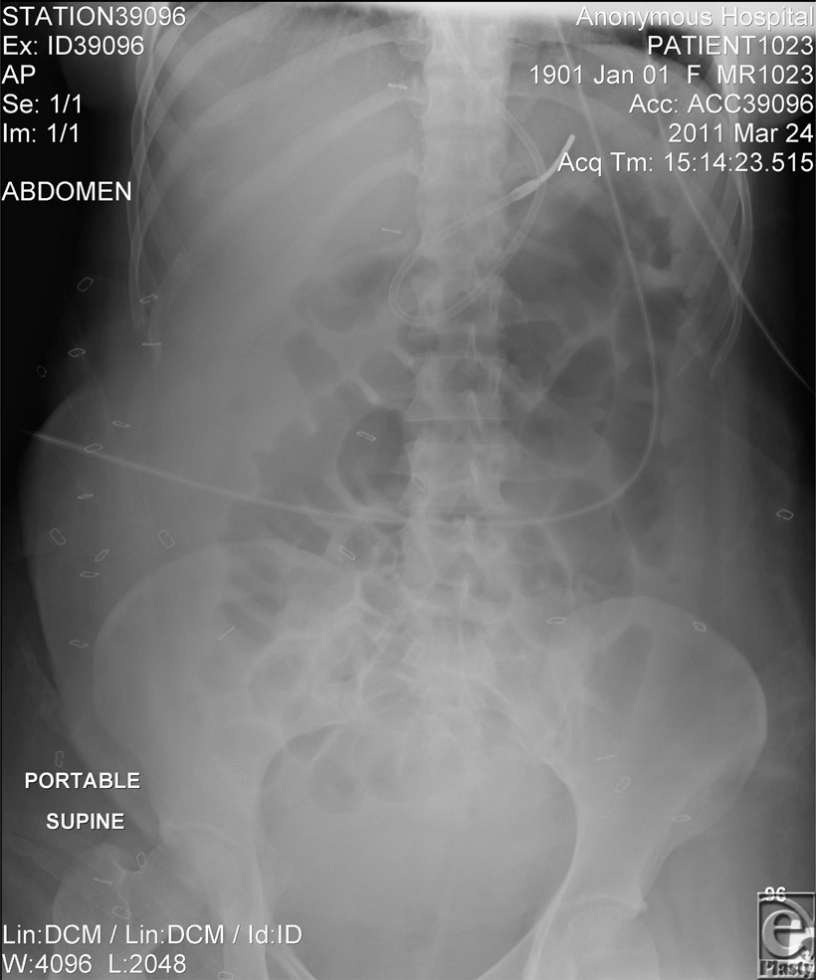
Taken a few hours after neostigmine administration. The reduction in colonic dilatation is clearly visible.

She was moved to the intensive care unit to monitor potential untoward bradycardia and 2 mg of neostigmine was administered intravenously over 4 minutes. The patient experienced immediate symptomatic relief. Approximately 30 minutes after the IV dose, all abdominal examination findings had returned to baseline. A follow-up abdominal x-ray ordered 3 hours later showed near-normal colonic profile, with a cecal diameter of 5 cm (Fig 2). No significant adverse effects were noted and she did not redevelop abdominal distension afterward.

**Figure 3:**
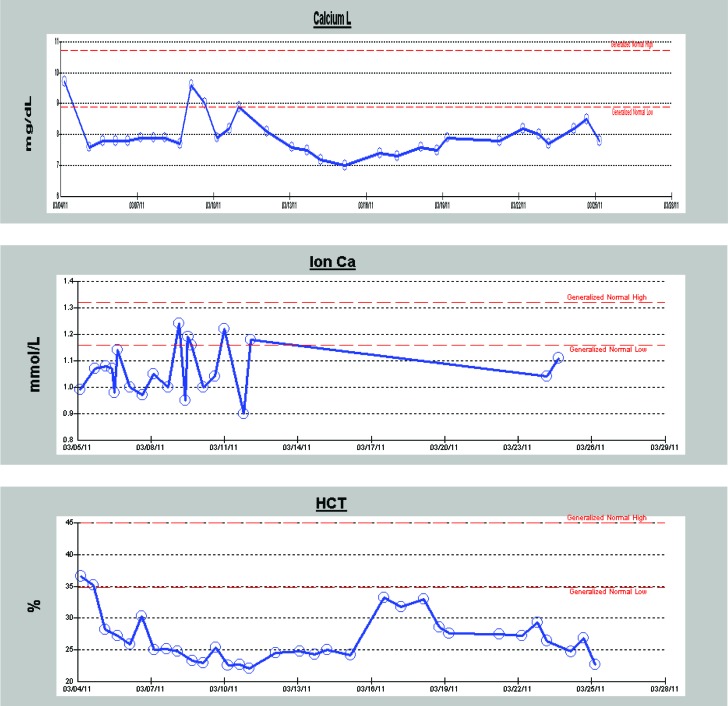
The red lines in these images represent the generalized normal high and low reference values for our laboratory. Serum albumin levels are not available for this patient. The slight bump in hematocrit, in the middle of the graph, on this patient represents the transfusion of packed red blood cells. The onset of symptoms occurs on or around 3/19/11 in these graphs. The administration of neostigmine occurs on 3/24/11 on these graphs.

Thankfully, over the past 2 years, we have not encountered a second case of ACPO in one of our burn patients. With an N of 1, however, it is difficult to draw any real conclusions about the precise cause of ACPO in this patient but we have identified numerous risk factors. As is common with large surface area burn patients, this patient was in the operating room for debridement and wound VAC changes every 3 days from admission until the time of this event for a total of 7 operations in that time span. All of her major electrolytes (calcium, magnesium, and phosphate) were low and required nearly daily repletion during the month leading up to her ACPO episode (Fig 3). In addition, she had an extremely low pain tolerance (something we commonly see in our younger patient population) and required multiple pain medications. She also had significant anemia that lasted for most of her stay and required a transfusion of 1 unit of packed red blood cells on hospital day 10, roughly 7 days prior to the onset of her abdominal symptoms. Her Dobhoff feeding tube was suboptimally placed and noted to be curled up into the fundus of the stomach which may have contributed to her abdominal discomfort. Other factors included frequent and sustained tachycardia, indwelling Foley catheter, *Escherichia coli* urinary tract infection, Pseudomonas aeruginosa wound infection (diagnosed the day following her neostigmine dose), waxing/waning fever over the course of her stay, and the presence of a triple lumen central line.

Her initial pain regimen consisted of the following: acetaminophen 650 mg tab by mouth every 4 hours, fentanyl 50-200 mcg IV per in-house pain-scale guidelines every 12 hours, hydromorphone 4 mg by mouth every 4 hours, oxycodone 30 mg sustained-release tab by mouth every 12 hours, morphine 2 mg IV every 4 hours, midazolam 1 mg IV every 4 hours, lorazepam 1 mg IV every 12 hours. Other medications included maintenance IV fluids dextrose 5% in 0.45% NaCl at 30 mL/h, and several nausea medications: prochlorperazine 5 mg IV every 6 hours, and scopolamine 1.5 mg transdermal. For her tachycardia, she was on metoprolol 12.5 mg by mouth every 12 hours.

Because gastrointestinal motility issues are common in our patient population, we start all patients on a standard bowel regimen. This initial conservative therapy consists of metoclopramide 10 mg IV every 8 hours, polyethylene glycol 3350 17 g by mouth every 12 hours, senna 1 tab by mouth daily, simethicone 1.2 mL by mouth daily, docusate 100 mg by mouth daily, esomeprazole 20 mg IV daily. In this patient's case, following the initial complaints of gastrointestinal discomfort and chest pain, a daily Fleets enema was added to her regimen.

The most striking lab abnormalities are presented here, with additional graphs provided in an addendum.

## METHODS

The literature search of PubMed (both for treatment consideration and for the purposes of this manuscript) was performed using the MeSH database. MeSH terms used were “Colonic Pseudo-obstruction,” “Burns,” and “Neostigmine.” These terms were used individually and in combinations. Major review articles and randomized controlled trials were chosen when available, with preference given to most recent publications. The best review articles were extensively cross-referenced for sources of additional information. However, we were unable to locate previous publications focused on the use of this therapy in burn patients, even though several articles noted that burn patients are at high risk for nonobstructive ileus.

## PATHOPHYSIOLOGY AND PROPOSED MECHANISMS

Despite improved knowledge of the pathophysiology of colonic dysmotility, the precise mechanisms underlying ACPO remain poorly understood. In summary, the following mechanisms may play a role in ACPO syndrome.

There are 2 opposing autonomic innervations that supply the gut. The parasympathetic system is responsible for activation of the digestive system, and its activation will increase gastric motility, gut secretions, and blood supply to the gut. The sympathetic system works opposite the parasympathetic system to decrease gut motility, gut secretions, and blood supply to the gut. The vagus nerve supplies parasympathetic innervation to the right colon, and the sacral spinal cord (s2-4) supplies parasympathetic innervation to the distal colon. The celiac and mesenteric ganglia supply the sympathetic innervation.^1^

While many theories have been proposed, ACPO remains a disease whose underlying pathogenesis is not yet completely understood. Animal models in which parasympathetic innervation was reduced, or sympathetic innervation was increased, have successfully mimicked ACPO. Some human cases have also demonstrated that a known imbalance of the parasympathetic and sympathetic systems will cause a patient to present with the symptoms of ACPO. If the parasympathetic innervations to the gut are reduced or removed, this will ultimately cause a substantial decrease or halt in gut motility, and massive colonic dilation ensues. In similar fashion, a substantial increase in sympathetic innervation to the gut, such as with systemic inflammatory response syndrome or chronic inflammatory conditions or even steroids can lead to ACPO. The previously discussed predisposing factors and comorbidities can cause this imbalance in the autonomic innervations.^2^

## TREATMENT OPTIONS

### Conservative treatment

In past studies, it has been demonstrated that in most cases, conservative treatment is extremely effective, and the majority of patients will resolve their symptoms within 3 days.^8^ If the patient does not improve with conservative treatment within 3 days, or if the cecum increases more than 12 cm, pharmacological treatment should be considered.

The major principle underlying conservative therapy is the identification and treatment of the underlying causative factors. Correction of electrolyte, fluid, and metabolic imbalances are vital. Patients should be kept *nil per os* until ileus resolves. Decompression of the stomach via nasogastric suctioning is recommended. Weaning of narcotics to the lowest possible dose that still relieves pain has been shown to be efficacious. Avoid anticholinergic agents, and other offending medications that may delay gastric intestinal motility. Frequent repositioning and ambulation of the patient has also been shown to be beneficial.^9^

In one study of cancer patients diagnosed with ACPO, each with other severe associated medical problems, 23 of 25 (92%) received conservative treatment only, resulting in successful resolution. One patient required surgery, and another died of multisystemic failure that was unrelated to ACPO. The mean cecal diameter of the 23 cancer patients was 11.7 cm, median time to resolution was 1.6 days, and the mean time to resolution was 3 days. The authors suggested that only in cases where the patient does not respond to conservative treatment within the time frame of 3 days should surgery or other invasive therapies be employed.^10^

### Pharmacological therapy

To our knowledge, the only pharmacological treatment that has been evaluated via a randomized, double-blind, placebo-controlled trial is IV neostigmine.^11^ In ACPO, specifically in burn patients, various treatment options have been described. However, the use of neostigmine as an alternative treatment modality does not appear to have been reported in the burn literature specifically.

Neostigmine is an acetylcholinesterase inhibitor and is reversible. It works on the basis of the theory that ACPO is caused by an imbalance between the parasympathetic and sympathetic nervous systems, namely a dysfunction in the parasympathetic system. When neostigmine is administered, acetylcholinesterase is inhibited. Acetylcholinesterase is a highly catalytic enzyme in the synaptic cleft that degrades the neurotransmitter acetylcholine, the neurotransmitter responsible for parasympathetic stimulation. When it is inhibited, acetylcholine is not hydrolyzed and remains in the synaptic cleft, resulting in an accumulation of neurotransmitter. The excess acetylcholine is then free to bind acetylcholine receptors, giving rise to increased parasympathetic stimulation and, in most cases, a resolution of the symptoms of ACPO.

In the randomized, double blind, placebo-controlled trial of neostigmine performed by Ponec et al,^11^ clinical response was defined as rapid reduction in abdominal distension and passing of flatus and/or stool. All patients exhibited a cecal diameter greater than 10 cm and had failed to respond to conservative treatment. Ten of 11 (91%) patients receiving neostigmine exhibited a clinical response, with a median response time of approximately 4 minutes, whereas none of the 10 patients receiving placebo responded. Open label neostigmine was provided to 7 patients in the placebo group and one in the neostigmine group who did not respond to initial infusion within 3 hours. All demonstrated swift decompression. Two patients who showed an initial response to neostigmine experienced a recurrence and then opted to undergo colonoscopic decompression. In total, 17 of 18 (94%) patients receiving neostigmine demonstrated a clinical response. In this study, the most common adverse effects of neostigmine were mild abdominal cramping, excessive salivation, and vomiting. Bradycardia and ensuing administration of atropine occurred in 2 of 19 patients (10.5%).^11^

### Mechanical decompression

A wide variety of nonsurgical techniques have been attempted to mechanically decompress the colon, including fluoroscopically guided decompression tubes; percutaneous cecostomy via endoscopic, laparoscopic, and surgical means; and colonoscopic decompression both with and without the use of decompression tubes. Of the nonsurgical invasive options for patients with substantial cecal distension lasting longer than 3 days, when neostigmine has failed or is contraindicated, the preferred method of choice is colonoscopic decompression based on the results reported in the available literature.^2^^,^^5^

Although no randomized clinical trials of colonoscopic decompression have been performed, a retrospective review of the cases (now totaling several hundred patients) reveals high success rates.^2^ In one large study looking at several series with more than 20 cases, success after initial treatment ranged from 61% to 78%.^5^ Rex reviewed literature for a total of 292 ACPO patients, treated via decompression colonoscopy, and estimated success after initial treatment at approximately 69%.^3^ Current literature also suggests that decompression tubes may be beneficial in lowering the recurrence rate.^8^^,^^12^ The safety of colonoscopic decompression in ACPO patients is evaluated via the occurrence of perforation, with the rate estimated at approximately 3%. In approximately 0% to 5% of patients, additional complications may arise, making this a very high-risk procedure, especially when compared to conservative and pharmacological therapy.^2^^,^^5^

### Surgery

If perforation and ischemia occur, or if the patient fails to respond to pharmacologic and endoscopic efforts, surgical management remains a viable option. Surgical management greatly increases morbidity and mortality and should therefore be employed only as a last resort. When perforation has not occurred, cecostomy remains the preferred option, as it is associated with a low morbidity and high success rate.^4^ Other surgeries include segmental/subtotal resection and exteriorization or primary anastomosis. These are recommended if perforation occurs.^2^

## CONCLUSION

Acute colonic pseudo-obstruction consists of massive dilatation of the colon in the absence of mechanical obstruction. This severe form of ileus, also known as “Ogilvie syndrome,” may develop in hospitalized patients and is associated with a wide variety of medical and surgical conditions. Prolonged bed rest, high doses of narcotic medications, sepsis, surgery, hypokalemia, and other electrolyte and metabolic imbalances are very commonly associated with development of ACPO. These predisposing conditions are all commonly associated with burn patients which make them particularly susceptible to developing ACPO. In ACPO, many treatment options have been described and used over the years. However, experience in using neostigmine for this indication in a burn patient does not appear to have been reported in the burn literature. Although large-scale clinical trials are not available, our review of the literature indicates that neostigmine is a safe and effective treatment option for nonobstructive ileus in hospitalized patients. This suggests that neostigmine may be a viable alternative to surgical options for ACPO in burn patients who have failed conservative therapy although better data in this population is necessary.
